# Exploring the Role of Long Noncoding RNAs in Breast Cancer Progression: A Comprehensive Study on Biomarkers and Therapeutic Approaches

**DOI:** 10.1002/cam4.71438

**Published:** 2025-12-29

**Authors:** Ramin Moeini, Amin AhsanAlkhat, Saeid Ghorbian

**Affiliations:** ^1^ Department of Biology Ta.C., Islamic Azad University Tabriz Iran

**Keywords:** breast cancer, LncRNA, metastasis, oncogene, prognosis, tumor suppressor

## Abstract

**Background:**

Among the many forms of cancer that plague women all over the globe, breast cancer (BC) is one of the most prevalent kinds. BC is a multifaceted disease that can manifest in many ways depending on the individual.

**Aims:**

The identification of new biomarkers that may be used to diagnose and prognosis of the BC, as well as those that can aid in the creation of novel therapeutic approaches and the understanding of how to regulate multiple pathways in metastasis, may initiate the process of creating novel therapeutic interventions for cancer patients.

**Materials and Methods:**

According to data from recent studies, long noncoding RNAs (LncRNAs) contribute to the development of BC via several distinct biochemical mechanisms. Remembering that long LncRNAs are longer than 200 nucleotides and do not encode proteins is essential.

**Results:**

Through its interaction with the EGF, TGF‐β, NF‐κB, PI3K/AKT, and p53 pathways, the abnormal production of LncRNA has a substantial impact on the progression of BC.

**Conclusions:**

This research investigates the biological properties of LncRNAs to ascertain their role in regulating cell signaling pathways and the subsequent changes in cell survival, invasion, and proliferation components that characterize BC.

## 1 Introduction

BC is the dominant kind of cancer that affects women all over the world, with 2.26 million reported cases and 685,000 deaths worldwide in 2020 [1]. The high mortality rate associated with BC is attributed to treatment resistance, tumor recurrence, and delayed detection [2]. Despite advances in medical research and treatment strategies, BC remains a leading cause of cancer‐related deaths among women. This highlights the urgent need for innovative approaches to improve early diagnosis, prognostication, and therapeutic outcomes. BC is not a single disease but a heterogeneous group of disorders with diverse molecular characteristics and clinical behaviors [3]. On the basis of gene expression profiles, breast tumors are categorized into several subtypes, including triple‐negative breast cancer (TNBC), HER2‐positive, and luminal A and B [3, 4]. For instance, because of the disease's aggressiveness and the scarcity of current treatments, TNBC poses significant therapeutic challenges. The result is a more dismal prognosis along with higher rates of invasion, metastasis, and recurrence compared to other BC subtypes. For this reason, it is crucial to comprehend the molecular pathways linked to TNBC pathogenesis and to identify novel treatment targets for these individuals [5]. This means that research is now being done on the potential use of LncRNAs as therapeutic targets for different malignancies and as biomarkers for diagnosis and prognosis [6]. According to recent genomic and bioinformatics investigations conducted across different species, eukaryotic genomes encode a diverse array of RNAs, including messenger RNAs (mRNAs) that code for proteins, short noncoding transcripts, and LncRNAs. In human cells, noncoding transcripts make up a more significant proportion of these RNAs. Although up to 70% of the human genome is transcribed, only 2% of those transcripts actually become proteins [7]. Genetic and molecular factors play crucial roles in the development and progression of BC. Among these factors, ncRNAs have garnered substantial interest. The remaining transcripts, known as ncRNAs, include a variety of functional RNA molecules that do not translate into proteins [8]. LncRNAs are a subclass of ncRNAs, typically longer than 200 base pairs and lacking an open reading frame (ORF), which prevents them from encoding proteins [9]. LncRNAs have been found in abundance by transcriptome sequencing and genome tiling array analysis. These analyses have revealed that LncRNAs possess intricate structures and origins. As a result, researchers believe that these molecules should be more than just classified based on their length and noncoding nature. Research indicates that LncRNAs share specific common characteristics. LncRNAs and coding proteins share similarities in chromatin states, such as the presence of H3K4me3 at promoters and H3K36me3 at transcribed regions [10]. Many common transcription factors control the expression of LncRNAs. RNA polymerase II is used to transcribe LncRNAs, which are often spliced via spliceosomes and include poly‐A tails, much like coding genes [11]. Research suggests that LncRNAs may influence numerous biological processes. These processes include cell differentiation, invasion, cell cycle, embryonic development, metastasis, and cancer progression. Evidence suggests a link between cancer‐related tumor development and metastasis and abnormal production of LncRNAs. These compounds are now being considered for use as potential therapeutic targets and biomarkers [12, 13]. Two types of LncRNAs play a significant role in the development of cancer of the breast: those that promote its progression and those that hinder its growth. Whether they encourage or obstruct BC's growth, their mode of action usually encompasses many dimensions, including but not limited to influencing BC's division and programmed cell death, molding drug resistance in BC, and influencing invasion in BC [11]. Notably, LncRNAs such as HOTAIR have shown promise in influencing cancer progression and may serve as valuable tools for therapeutic intervention. Through their ability to modulate the production of both mRNAs and miRNAs, these molecules may mechanistically affect cancer signaling networks [14, 15]. Despite the progress in understanding LncRNAs' roles, the complexity and variability of their functions necessitate further research. Identifying specific LncRNAs involved in BC and elucidating their mechanisms of action could lead to novel diagnostic and therapeutic strategies. Therefore, exploring the expression patterns and functional roles of LncRNAs in BC subtypes, particularly in aggressive forms like TNBC, is crucial for advancing personalized medicine and improving patient outcomes. Most LncRNAs have tissue‐specific expression. Just a tiny percentage of the more than 200 different kinds of LncRNAs that have been studied relatively thoroughly so far have been shown to work in vivo; many have only been proven to function in vitro [11, 16]. This investigation aims to determine which LncRNAs have the most impact and role in the formation of BC.

## 2 Categorization of LncRNAs in Genomic Sites

**FIGURE 1:**
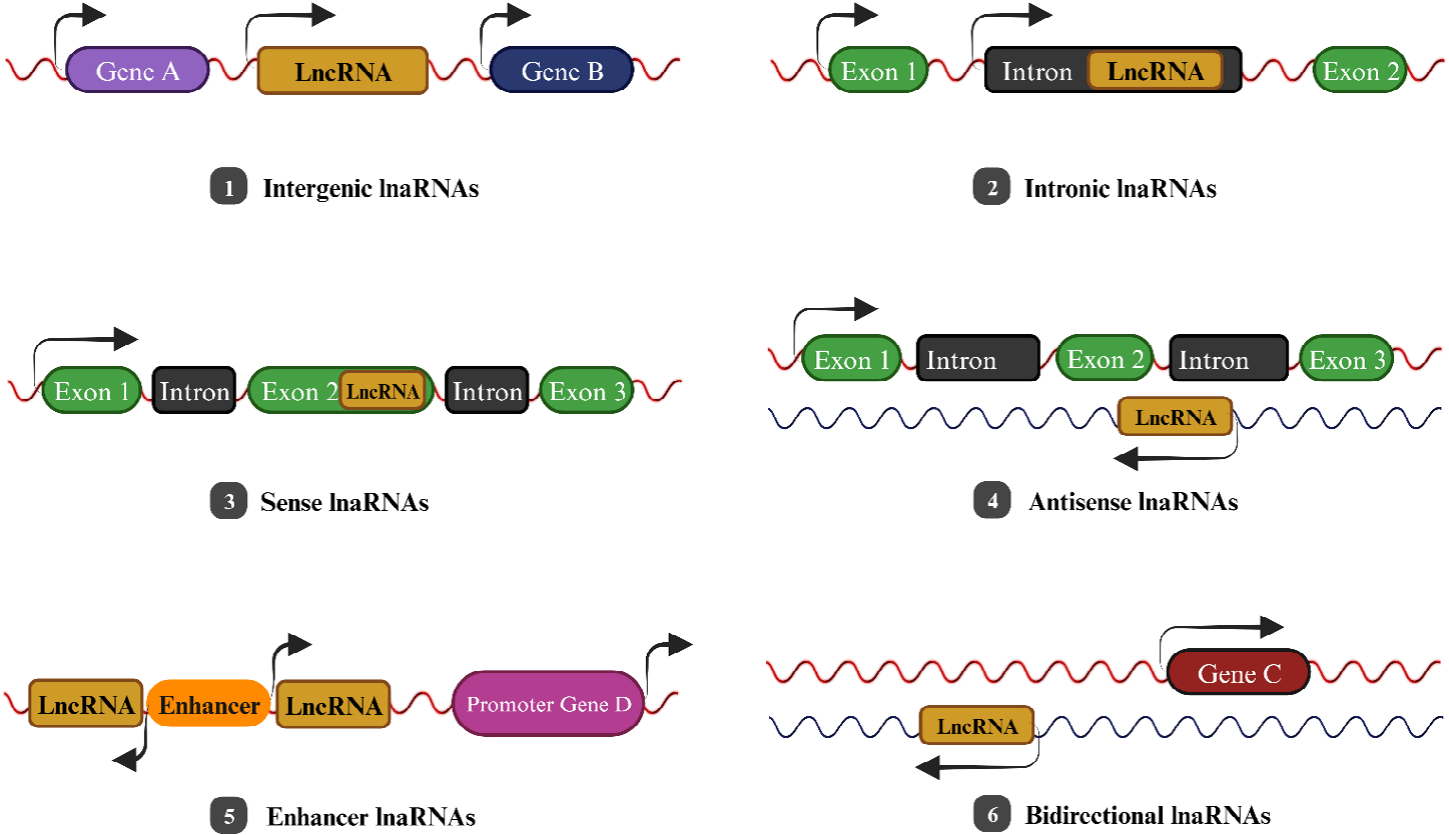
Categorization of LncRNAs according to the genomic sites of their transcripts.

LncRNAs play a significant role in many things that happen in people, both usually and abnormally [17]. In order to differentiate LncRNAs from other kinds of RNAs and to comprehend the functional significance of LncRNAs, it is necessary to have a comprehensive knowledge of the biogenesis of LncRNAs [18]. Although the synthesis of LncRNAs is controlled by stimuli that are particular to cell kinds and stages, it is specific to distinct cell types and stages. Eukaryotic genomes include a variety of DNA elements that are used to transcribe LncRNAs. These DNA elements include enhancers, promoters, and intergenic regions [19]. LncRNAs can be found in the cytoplasm or nucleus of a cell. Depending on where they are located, they can act nearby (cis) or far away (trans) to regulate gene expression [13]. As we can see from the diagram below (Figure 1), LncRNAs can be categorized into six different groups based on their characteristics, gene loci, and interactions with neighboring genes. Protein‐coding genes are separated from one another by DNA sequences that make up intergenic LncRNAs [9]. Localized noncoding RNAs are found inside the introns of genes that code for proteins [5]. Many protein‐coding genes contain sense LncRNAs inside their exons and introns. Antisense LncRNAs follow a different transcriptional pathway than genes encoding proteins [20]. A region known as a promoter enhancer is where enhancer LncRNAs are produced. On the opposite strand, near a coding transcript, there are bidirectional LncRNAs [9]. LncRNAs may impact the significant molecular process underlying BC: the alteration of cell cycle control (Table 1) [21]. According to recent studies, LncRNAs can regulate gene expression through various methods, such as altering chromatin structure, controlling transcription (Figure 1) [20], and affecting post‐transcriptional processes. Furthermore, it has been demonstrated for many human tumors that these LncRNAs directly affect cancer cells' metastasis in vivo and in vitro [22]. LncRNAs regulate several signaling pathways linked to the metastasis of BC. If these signaling pathways are compromised, BC cells have the potential to infiltrate nearby tissues and spread to other organs [23].

**TABLE 1:** Instances of various categories of LncRNAs as per the categorization shown in Figure 1.

LncRNAs types	LncRNA example
Intergenic LncRNAs	MALAT1, LINC‐ROR, and ANCR [9].
Intronic LncRNAs	PRUNE216, PCA317, COLDAIR18, SYISL19 [5].
Sense LncRNAs	GAS513, ecCEPBA20 [20].
Antisense LncRNAs	TALAM121, PDCD4‐AS122, Wrap5323 [20].
Enhancer LncRNAs	LEENE24, SLC4A1125 [9].
Bidirectional LncRNAs	LINC0044126, Catsper1au27 [9].

## 3 Biological Roles of LncRNA

Many studies have demonstrated that LncRNAs are involved in the pathogenesis of various cancers, including BC, one of the most common diseases. The role of LncRNAs in the development of BC is not just limited to the early stages of the disease. They continue to exert their influence as the disease progresses, contributing to the abnormal production of these RNAs [24]. BC is a complex disease, and a multitude of signaling pathways is involved in its progression. Recent studies have revealed that LncRNAs are not passive players in this process. They actively control and trigger specific signaling pathways that are crucial for the proliferation of BC cells [25, 26, 27, 28, 29]. Approximately 90% of cancer‐related deaths are caused by the intricate process of malignant invasion and metastasis. The term “metastasis” describes the process by which primary tumor cells travel through the blood or lymphatic systems, eventually arriving at distant secondary organs and then proliferating. This chapter examines the significant role that LncRNAs play in the pathways they act in [23, 30, 31, 32, 33, 34, 35]. Apoptosis is a highly regulated process of cell death essential for the proper development of tissues. When apoptosis is dysregulated, tumorigenesis proceeds more efficiently. Cellular death is triggered by various LncRNAs [29]. Through interactions with several pathways, including TGF‐β, Hippo, STAT3, Hippo, NF‐κB, p53, PI3K/AKT, and EGF, the uneven expression of LncRNA significantly impacts the spread of BC [9]. Differences in each patient's pharmaceutical reaction complicate the treatment of breast cancer (BRCA); therefore, predicting responses might help in treatment planning. Learning more about the fundamental biological processes underlying drug responses might enhance the effectiveness of chemotherapy and the results of BRCA treatment (Table 2) [63]. Recent research suggests that LncRNAs may play a role in controlling treatment resistance in BC by explicitly targeting particular genes [20]. The primary mechanism by which LncRNAs contribute to the emergence of drug resistance is their capacity to alter gene expression patterns [64]. LncRNA H19 is a downstream protein of ERγ in BC. It has been noted that in BC cells expressing estrogen receptors (ER), H19 decreases the apoptotic response and increases resistance to paclitaxel. This effect is accomplished by inhibiting NOXA and BIK transcription. H19 inhibits the BIK protein that relies on EZH2 epigenetically [65].

**TABLE 2:** Identification of LncRNAs in the BC and their potential roles [24, 36, 37, 38, 39, 40, 41, 42, 43].

LncRNA	Abbreviation	Size (kb)	Location	Expression	Tumor suppressor/oncogene	Biology functions of LncRNA	Signaling pathway	Cancers of the breast subtypes	Function in breast cancer	References
HOTAIR	HOX antisense intergenic RNA	2.2	12q13.13	Upregulated	Oncogenic	Cell invasion and metastasis	TGF‐ꞵ pathway with stimulatory role [42]	ER+/HER2+/TNBC	Association with transcriptional factor/molecular scaffold; epigenetic gene silencing	[36]
MALAT 1	Metastasis‐associated lung adenocarcinoma transcript 1	8.7	11q13	Dysregulated	Oncogenic	Cell proliferation/Cell invasion and metastasis	miR101‐3p/mTOR/PKM2 pathway with Inhibitory Role	ER+/TNBC	The levels of miR‐101‐3p showed an inverse relationship with the expressions of MALAT1 and the inhibited BC cells. In addition, miR‐101‐3p directly targeted the mTOR/PKM2 pathway. Overexpression of MALAT1 led to a significant decrease in gene levels of miR‐101‐3p and an increase in protein expressions of the mTOR/PKM2 pathway. The inhibition of miR‐101‐3p resulted in the blocking of MALAT1.	[37, 38, 40]
H19	H19	2.3	11p15.5	Upregulated	Oncogenic	Cell invasion, metastasis and Cell proliferation	AKT pathway with stimulatory role/TGF‐ꞵ pathway with stimulatory role	ER+	H19 has the potential to activate the canonical Wnt/β‐catenin signaling pathway and modulate the miR‐340‐3p/YWHAZ axis, which in turn might lead to metastasis and EMT.	[41, 43]
GAS5	Growth arrest‐specific 5	0.6–1.8	1q25.1	Downregulated	Tumor suppressor	Cell apoptosis	FOXO1/PI3K/AKT pathway with inhibitory role	HER2+/TNBC	When GAS5 is activated, it leads to cell death and inhibits cell growth and spread by interacting with miR‐196a‐5p. This interaction has an impact on the PI3K/AKT/FOXO1 signaling pathway.	[39, 44, 45]
XSIT	X inactive‐specific transcript	19	inactive X‐chromosome	Downregulated	Tumor Suppressor	Inhibited cell proliferation, EMT and induced apoptosis	miR‐454 via targeting AKT pathway	TNBC	miR‐454 was identified and verified as an important target of XIST. A rescue test shown that increasing the expression of miR‐454 might counteract the anti‐tumor effects of XIST restoration on TNBC cells.	[46]
BC200	Brain cytoplasmic 200	13	2p21	Upregulated	Oncogenic	Cell proliferation, migration, and invasion	—	ER+/TNBC	Stimulates the spread of cancer by facilitating invasion	[47, 48]
ZFAS1	Zinc finger antisense 1	17.5	20q13.13	Downregulated	Tumor suppressor	Cell invasion/metastasis	PTEN/PI3K/protein kinase B (AKT) pathway/STAT3 pathway	TNBC	The upregulation of ZFAS1 facilitates the cessation and it induces apoptosis in breast cancer cells via modulating the cell cycle. Furthermore, it has been shown that ZFAS1 has a role in controlling the STAT3 pathway, cellular proliferation, and the spread of cancer cells in triple‐negative breast cancer (TNBC) cells.	[49, 50]
CCAT2	Colon cancer associated transcript 2	—	8q24.21	Upregulated	Oncogenic	Cell proliferation	Wnt pathway with stimulatory role	ER+/TNBC	Cell proliferation was significantly impeded and cancer cells exhibited an enhanced stem‐like phenotype when cytoplasmic CCAT2 was present in vitro within the luminal subtype of MCF‐7 or T47D cancer of the breast cells. The stimulation of miR‐221‐p27 signaling resulted in the suppression of tumor development in vivo. On the other hand, OCT4‐PG1 expression increased and cancer cell stemness was stimulated as a consequence of overexpressed CCAT2 in the nucleus.	[51, 52]
SPRY4‐IT1	Sprouty4‐intron transcript 1	—	5q31.3	Upregulated	Oncogenic	Abnormalities in the proliferation of cells, less invasion, and more apoptosis	Wnt/β‐catenin signaling pathway	ER+	The protein SPRY4 IT1 has a crucial role in promoting the growth and differentiation of cancer of the breast cells, as well as improving the capacity of breast cancer stem cells to regenerate and maintain their stemness. The upregulation of TCF7L2 expression is accomplished by targeting miR 6882 3p.	[53]
ANCR	Anti‐differentiation noncoding RNA	0.85	4q12	Downregulated	Tumor Suppressor	Cell invasion and metastasis	TGF‐ꞵ pathway with Inhibitory Role	ER+/TNBC	Metastasis mainly through decreasing EZH2 stability.	[54, 55]
PVT1	Plasmacytoma variant translocation 1	300	8q24	Upregulated	Oncogenic	Inhibit apoptosis/Cell proliferation	Promotes KLF5/beta‐catenin signaling	TNBC	Long noncoding RNA A study has found that PVT1 plays a crucial role in the development of triple‐negative breast cancer (TNBC) by promoting the signaling pathway involving KLF5 and beta‐catenin. Through the action of EZH2, PVT1 was found to silence the expression of FOXF1, leading to increased cell proliferation and decreased apoptosis in breast cancer.	[56, 57]
UCA1	Urothelial carcinoma associated 1	1.4	19p13.12	Upregulated	Oncogenic	Cell invasion and metastasis	Wnt pathway with inhibitory role	HER2+	UCA1 has the ability to regulate the process of EMT in MDA‐MB‐231 cells. When UCA1 is suppressed, it negatively affects the mesenchymal characteristics of these cells. The overexpression of UCA1 enhances the invasiveness of cancer of the breast cells, maybe via the activation of the Wnt/β‐catenin signaling pathway.	[58]
LINP1	LncRNA in nonhomologous end joining pathway 1	—	10p14	Upregulated	Oncogenic	Cell apoptosis	P53 pathway with inhibitory role	TNBC	LINP1 enhances epithelial‐mesenchymal transition (EMT) via impeding the P53's anti‐metastatic actions.	[59, 60]
SRA	Steroid receptor RNA activator	0.7–1.5	5q31.3	Upregulated	Oncogenic	Cell apoptosis and Cell proliferation	p38 MAPK, Notch and TNFα pathways	ER+	SRA enhances the activity of ERα by activating the MAPK signaling pathway and phosphorylating the serine residue at position 118 in ERα via the AF‐1 activity of the receptor.	[61]
LINK‐A	Long intergenic noncoding RNA for kinase activation	1.5	1q43	Upregulated	Oncogenic	Cell proliferation, apoptosis, cell invasion and migration	AKT and HIF1α signaling	TNBC	LINK‐A plays a crucial role in activating HIF1α signaling, especially in TNBC, contributing to tumor development. As a result of its interaction with PtdIns P3, a lipid protein that is part of cell signaling pathways, the AKT pathway becomes overactive.	[62]

## 4 Action and Functions of LncRNAs

**FIGURE 2:**
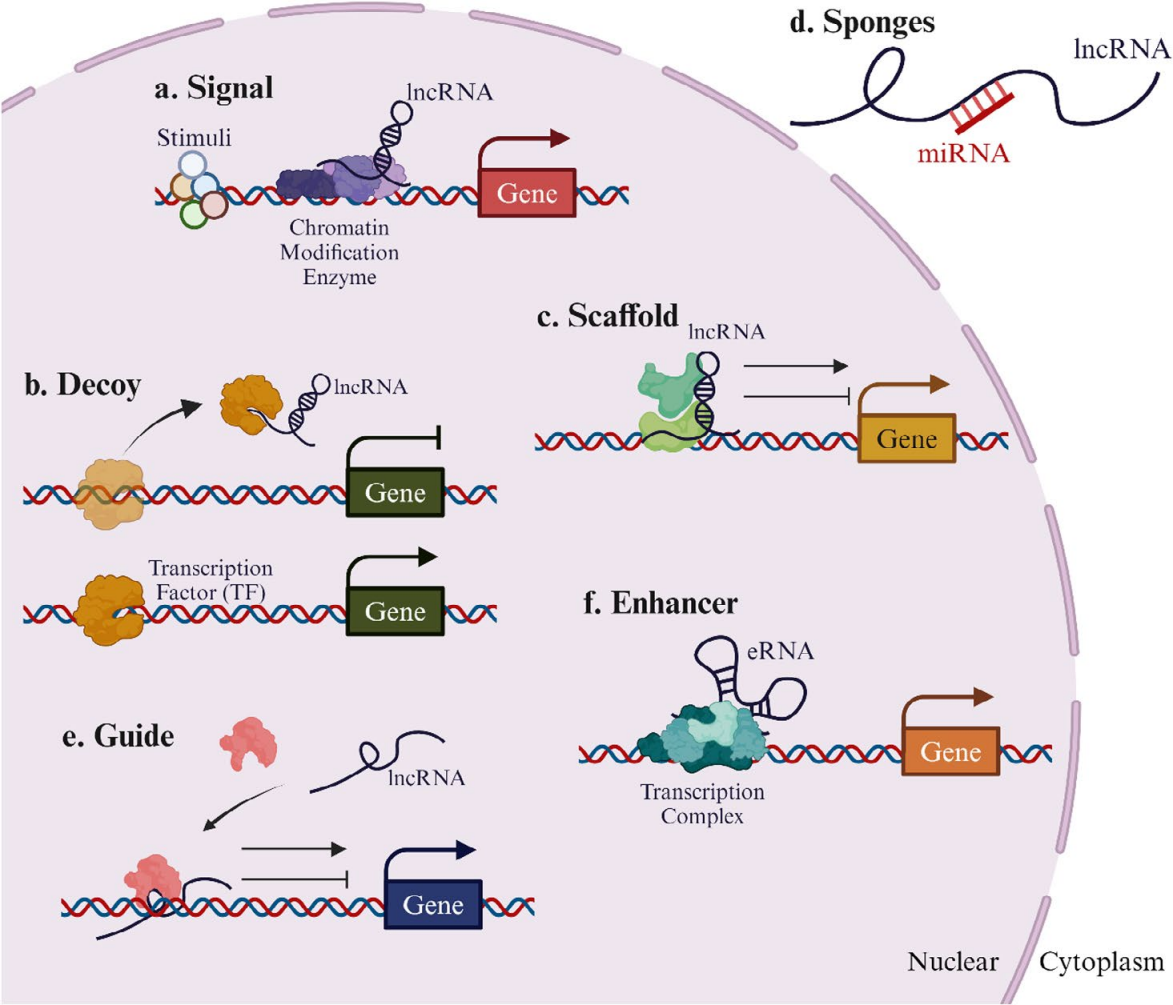
Transcriptional‐level functional processes of LncRNAs. (a) Signal LncRNAs: LncRNAs receive a signal when stimulated, directing them to engage with chromatin‐modifying enzymes. This interaction ultimately impedes the transcription process. (b) LncRNAs that serve as decoys have a heightened affinity towards certain regulatory factors. The interference of regulatory factors with DNA binding is caused by the binding of LncRNAs, leading to the initiation of transcriptional repression. (c) Scaffold LncRNAs significantly contribute to the creation of RNA‐protein (RNP) complexes. These proteins primarily serve the purpose of regulating transcription via the activation or inhibition of specific target genes. (d) LncRNAs absorb the miRNAs, hence impeding the gene suppression caused by miRNAs. (e) LncRNAs are essential in regulating chromatin and organizing transcription factors at particular genomic locations. (f) Contacts of chromatin are subject to the impact of an enhancer, which can be expressed through an enhancer RNA (eRNA).

**FIGURE 3:**
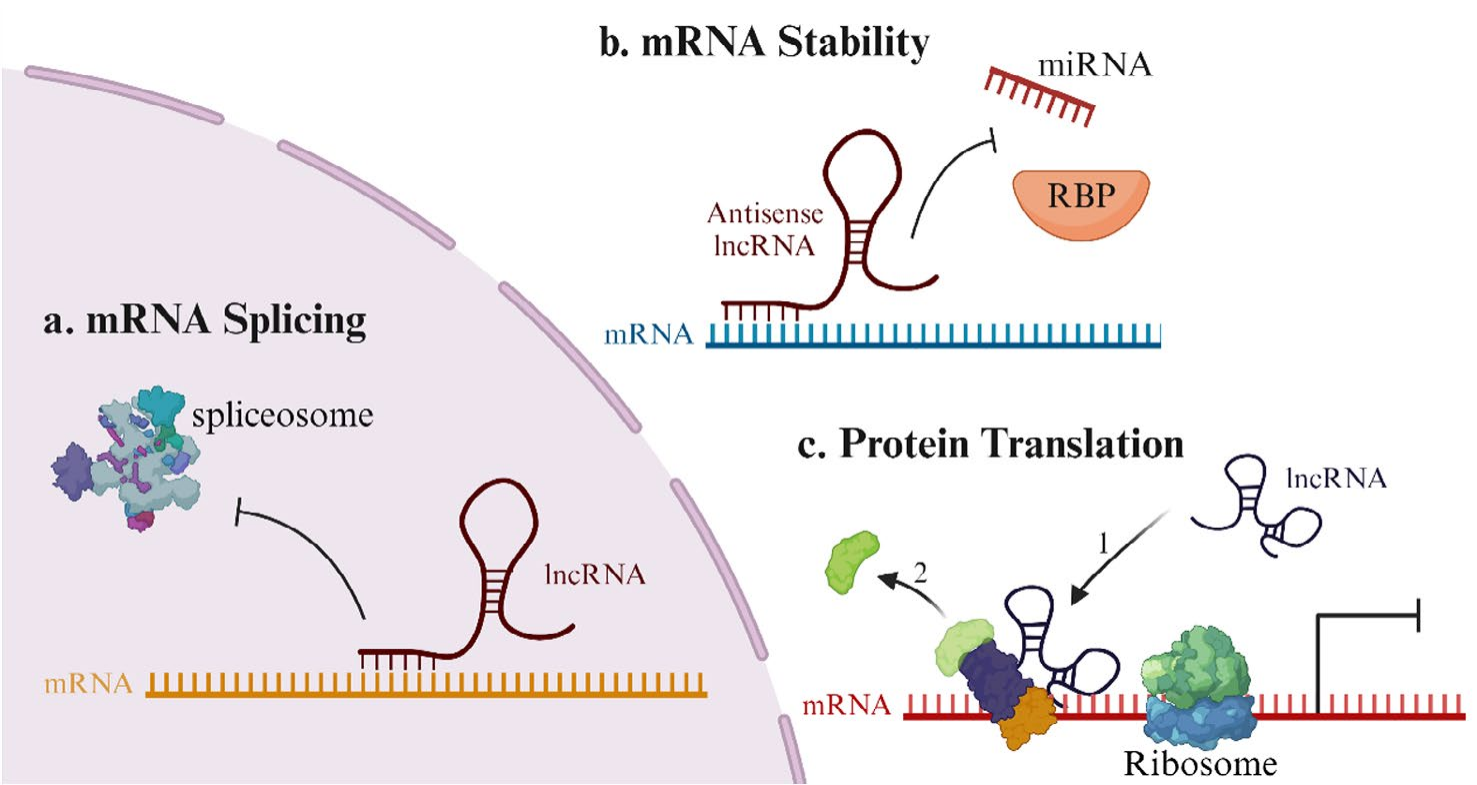
LncRNAs at posttranscriptional levels are responsible for their functional processes. (a) In order to regulate the phosphorylation forms of serine/arginine splicing factors, LncRNAs are responsible for modulating the splicing arrangement of pre‐mRNA molecules. (b) LncRNAs enhance the stability of mRNAs by creating RNA duplexes, inhibiting degradation. (c) LncRNAs influence protein translation by facilitating the recruitment of the translation‐related protein complex.

LncRNAs are critical as therapeutic agents and prognostic and diagnostic indicators for BC [66]. Comprehensive knowledge of the various processes by which LncRNAs control metastasis could result in the development of innovative treatment modalities for cancer patients [24]. LncRNAs are categorized based on their impact on methylation and chromatin state, the stability of proteins and complexes, and their ability to influence the spread of cancer [67]. LncRNAs function in three stages of biological processes: transcription, epigenetic, and post‐transcriptional, when they lack an open reading frame (ORF). LncRNAs can be used as a (1) signal or a (2) decoy to change the number of transcriptional genes produced [68] (Figure 2a,b). Transcription of LncRNAs occurs when specific conditions activate their DNA segments. In addition to acting as biological event markers, conditional transcripts such as XIST and HOTAIR can send additional signals. The decoy's RNA motif, which contains the genes GAS5 and MALAT1, is similar to the matching DNA motif. They serve only as scapegoats for proteins that bind DNA. That is their only purpose. (3) To regulate gene expression, LncRNAs act as scaffold elements, building chromatin‐modifying structures at particular sites. The LncRNAs can interact with transcription factors and other transcriptional regulators simultaneously (Figure 2c) [69]. (4) Often referred to as competitive endogenous RNA, LncRNAs function as sponges to reduce the effect of microRNAs on the genes they target [70]. In malignant breast tumors, LncRNAs may absorb miRNAs as they are competing endogenous RNAs (Figure 2d). (5) Guide LncRNAs facilitate the active or passive expression of genes by relocating regulatory elements (Figure 2e) [71]. (6) Enhancer regions (ERs), which are mediated by enhancer RNAs (eRNAs), allow an enhancer to modify the “chromatin interaction” of DNA. According to one theory, these LncRNAs are attached to ERs rather than released from them (Figure 2f) [72]. LncRNAs perform post‐transcriptional control of mRNA splicing, stability, and protein translation. (1) LncRNA has an impact on the post‐transcriptional process of regulating RNA splicing (Figure 3a) [73]. Because LncRNAs are antisense RNAs that can also impact a known indication of mRNA stability, they significantly contribute to the spread of BC (Figure 3b). How LncRNAs regulate the vital process of protein translation is shown in (Figure 3c) [74]. LncRNAs are crucial for post‐transcriptional control because they support several activities, including splicing, protein translation, and mRNA stability [75, 76]. DNA methylation occurs in a gene's downstream enhancer region, and LncRNAs play a crucial role in controlling this methylation, which silences the gene epigenetically [77]. Histone modification factors work in concert with LncRNAs to modify histones' methylation, acetylation, or ubiquitination [78]. LncRNAs have an impact on DNA methylation and histone modifications. They are essential regulators of gene expression because they alter the recruitment or sequestration of epigenetic factors [79, 80]. The modification of epigenetic expression is recognized as a hereditary characteristic that endures due to chromosome changes instead of DNA sequences. Researchers have focused much of their attention on two epigenetic modifications: DNA methylation and histone alteration. Both are characterized by their reversibility and dynamic control [81]. Tumor growth has been linked to many LncRNAs cooperating with enzymes that methylate DNA or change histones to improve gene transcription [82, 83]. (3) To control the transcriptional activity of specific genes, LncRNAs can directly engage with chromatin modification complexes to drive the rebuilding or conformational modifications of chromatin [84]. Chromatin remodeling is crucial in regulating gene transcription in eukaryotic organisms like bacteria because it may help move chromatin from condensed to loose states. The specific subunits responsible for ATP hydrolysis allow us to distinguish between the three primary types of chromatin remodeling complexes. Numerous complexes, such as the SWI/SNF and ISW complexes, have been studied. Numerous LncRNAs have been demonstrated to be involved in the chromatin remodeling processes that the SWI/SNF pathway enables as malignancies progress. LncRNAs can draw chromatin remodeling complexes to the region of the target locus that drives expression, such as ISW, INO80, and SWI/SNF. This process occurs, and it causes a change in the chromatin structure. This modification increases gene activation by making genes more accessible to tightly packed DNA through transcription factors. On the other hand, specific LncRNAs can start gene repression by separating chromatin remodeling complexes from the particular cis areas they target [85, 86, 87].

## 5 Oncogenic and Tumor‐Suppressive Roles of LncRNAs in BC

LncRNAs are now known to be involved in several physiological and cancer‐related functions such as invasion, proliferation, apoptosis, and carcinogenesis. Even with tremendous advancements in our knowledge of the molecular mechanisms underlying BC and the creation of specialized therapies based on four molecular kinds, we are still unable to reduce the disease's high incidence and fatality rates effectively. Multiple studies have discovered that many LncRNAs significantly correlate with BC and demonstrate a robust association with it [88, 89, 90].

### 5.1 HOTAIR

**FIGURE 4:**
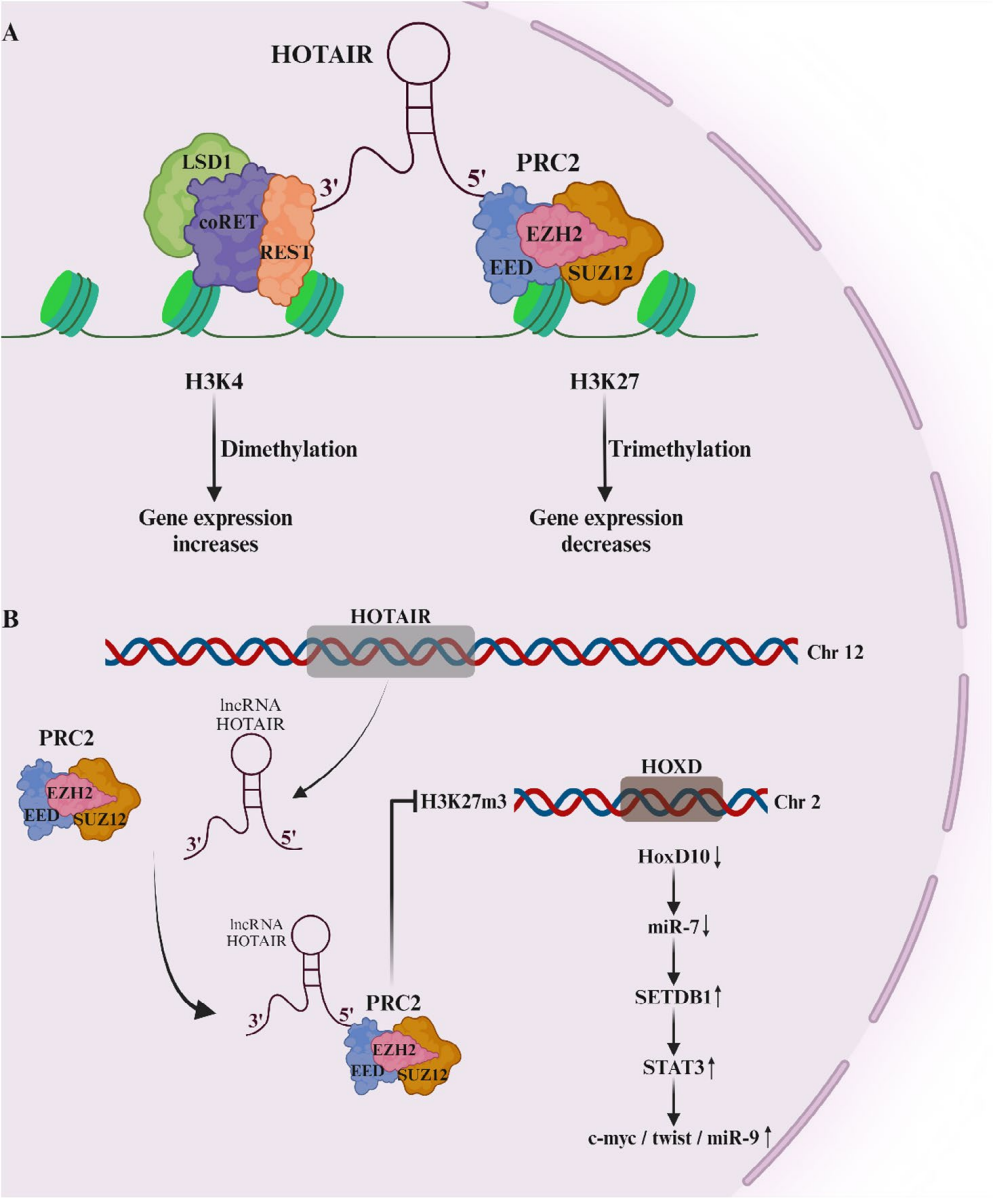
(A) Scaffold HOTAIR functions by forming a complex with either the PRC2 complex or the CoREST/LSD1/REST complex, resulting in the control of genes through histone changes. (B) HOTAIR directs the PRC2 complex to suppress the HoxD10 locus by epigenetic repression, thus reducing the levels of miR7. The downregulation of miR7 leads to suppression.

Using a tiling array, the LncRNA known as HOTAIR was successfully discovered for the first time in 2007 [91]. The LncRNA is at 12q13.13, the HOXC locus. It has 2158 nucleotides. This entity's principal purpose is to momentarily obstruct the HOXD gene's transcription process located on chromosome 2 [92]. The findings showed that the primary BC had a higher concentration of HOTAIR than the surrounding noncancerous tissue. It was previously demonstrated that HOTAIR promoted the growth of cancer cells and could serve as a strong prognosticator [93, 94, 95]. Numerous research studies have examined HOTAIR's mechanism, demonstrating its function as a scaffold for binding multidomain functional complexes. The PRC2 subunits, which include SUZ12, EDD, and EZH2, are engaged by the 5′ terminus of HOTAIR. Because of this relationship, H3K27 is more likely to be dimethylated, which causes HOTAIR to silence specific genes. Moreover, the 3′ terminus of HOTAIR interacts with the REST/CoREST/LSD1 complex, promoting H3K4 demethylation and triggering the expression of genes [96, 97] (Figure 4A). Thus, HOTAIR modifies specific histone codes, affecting several downstream genes [98]. It was concluded that most of these genes were associated with the developmental and cellular signaling pathways based on the results of the Gene Ontology research. A study conducted by an independent investigator revealed that HOXD interacts with the promoter of miR‐7 to raise it. Therefore, because miR‐7 has a sequence complementary to the 3'UTR of SETDB1 mRNA, it can mute SETDB1 and the STAT3 pathway. HOTAIR inhibits HOXD [42] in regulating these activities (Figure 4B).

### 5.2 MALAT1

**FIGURE 5:**
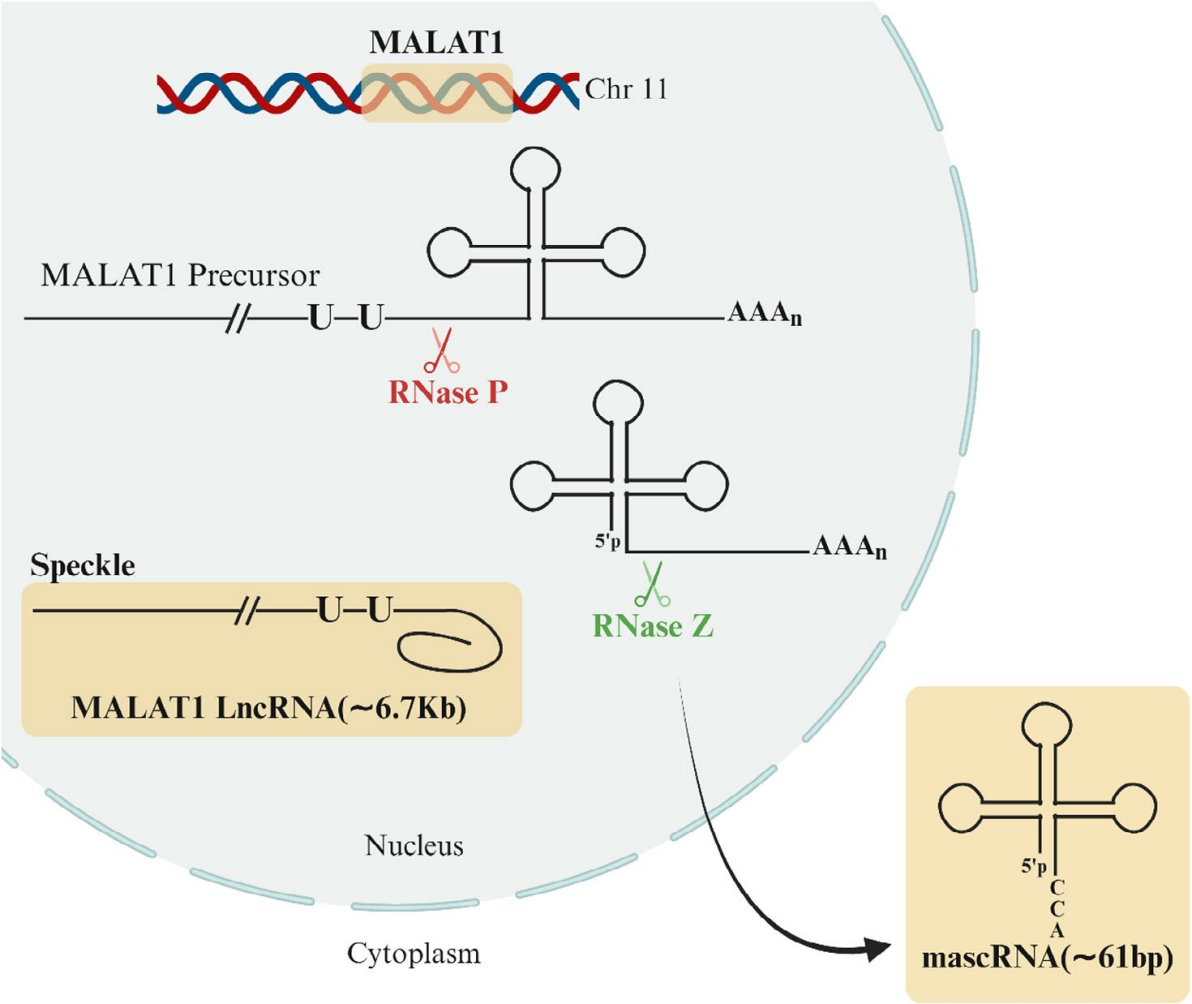
3′ end of MALAT1 and the 5′ end of mascRNA are formed due to the cleavage of the MALAT1 precursor by RNase P, which occurs immediately after the poly(A)‐rich site. CCA is then added to the mixture after the 3′ end of mascRNA has been cleaved by RNase Z during the subsequent step. The MALAT1 protein exhibits nuclear speckle accumulation, whereas the mascRNA is transported to the cytoplasm.

**FIGURE 6:**
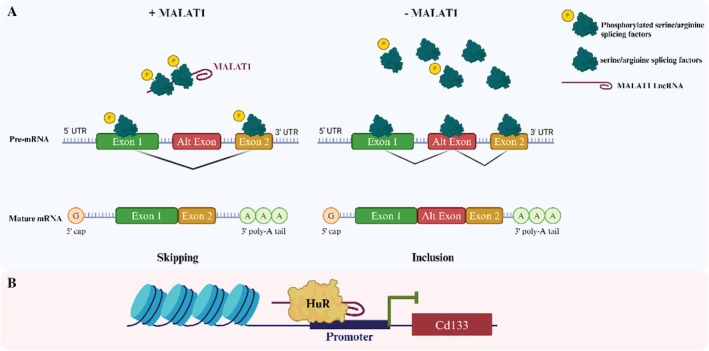
LncRNAs modulate the splicing arrangement of pre‐mRNA molecules through the regulation of phosphorylation forms of serine/arginine splicing.

MALAT1 is located in the 11q13.1 region and has a total length of 8.7 kilobases. The original transcript undergoes cleavage to yield two distinct components: a mature 6.7 kilobase transcript with a brief poly (A) tail that remains in the nucleus and a mature 61‐nucleotide tRNA‐like mascRNA, whose exact role is yet unclear. Both of these components are subsequently transported to the cytoplasm [99]. An early discovery revealed that the gene MALAT1 was linked to the development of metastases in patients with non‐small‐cell lung cancer. A significant body of recent studies has revealed that MALAT1 is raised in various cancer types, including BC. Nuclear MALAT1 significantly influences the processes above (Figure 5) when cancer cells proliferate, invade, and migrate [100, 101]. One essential role of this control of pre‐mRNA alternative splicing is its interaction with serine/arginine (SR) splicing factors (SF). In nuclear speckles of healthy cells, several SR proteins are hyperphosphorylated. On the other hand, in cellular systems where MALAT1 has been reduced, there is a noticeable increase in the number of cellular SR proteins as well as the percentage of dephosphorylated SR proteins to phosphorylated SR (Figure 6A) [102]. Furthermore, it was interesting that MALAT1 forms a restrictive complex with the RNA‐binding protein HuR. Inside this complex, HuR binds to the promoter of the CD133 gene, exerting a negative regulatory effect on the gene's expression (Figure 6B). CD133 stimulates the EMT pathway and is a stand‐in for cancer stem cells in various cancer types [37, 103].

### 5.3 H19

**FIGURE 7:**
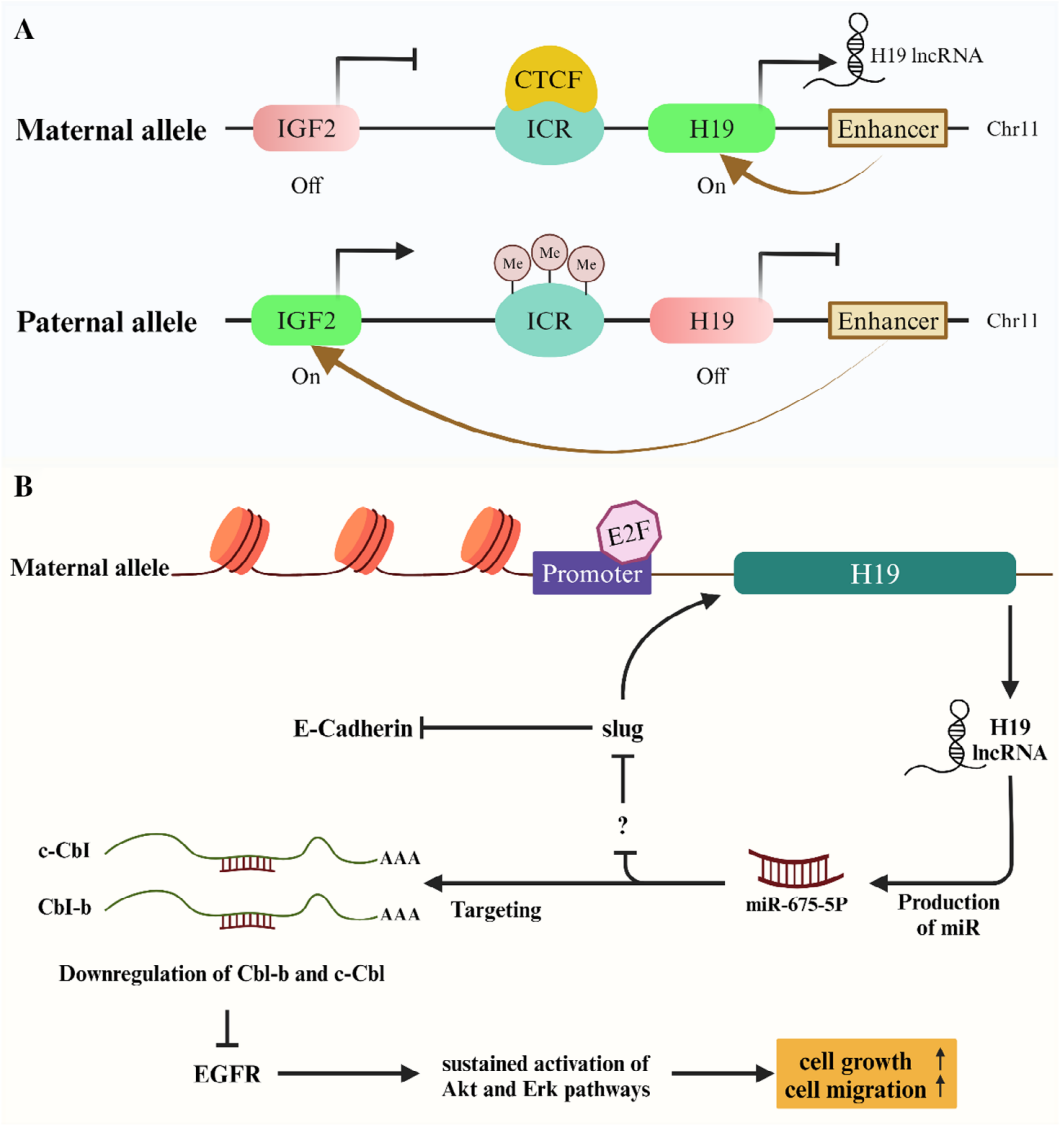
Conversely, the expression of Igf2 is only seen in the paternal allele, while the expression of H19 is exclusively observed in the maternal inheritance pattern. This pair of genes possesses an enhancer and imprinting control regions (ICR). The methylation of the paternal ICR inhibits the interaction between the CTCF protein and the ICR. Ultimately, the paternal Igf2 enhancer and promoter may interact, allowing for the transcription of Igf2. Conversely, the CTCF protein can connect with the un‐methylated maternal ICR. Consequently, this hinders the interaction between the enhancers and the Igf2 promoter. This particular allele exhibits exclusive transcription of the H19 gene. H19 serves as the precursor for miR‐675‐5p, specifically targeting the mRNA of Cbl‐b and c‐Cbl in breast cancer. miR‐675‐5p is released by the H19 gene's exon‐1. Cell growth and migratory potential are enhanced when the Akt and Erk pathways are persistently activated, which is caused by reducing the expression of the Cbl‐b and c‐Cbl proteins [111, 112].

The first LncRNA H19 is essential for genomic imprinting throughout development and growth [104]. The gene, located 200 kb from the IGF‐2 gene in the 11p15.5 region, encodes a 2.3 kb LncRNA [105]. Splicing, polyadenylation, capping, and translocation of the transcript into the cytoplasm, which links with polysomes, are all part of this process [106]. A single paternal allele is expressed by the H19 gene, categorized as an imprinting gene [107]. Although only the mother expresses H19, the paternal allele produces IGF‐2 [108]. A wide range of cancer types exhibit increased expression of H19, including ovarian serous epithelial carcinoma, stomach, lung, breast, bladder, esophageal, and so forth [109, 110]. On the other hand, its significant contribution to tumor invasion and angiogenesis is indicated by its involvement in controlling the expression of genes linked to metastasis and blood vessel formation (Figure 7A) [106]. According to the study, LncRNA H19 was produced at higher levels in BC cells than in MDA‐MB‐231 cells [104, 113]. Like a microbiologist, a transcription factor is essential for controlling the expression of specific genes. E2F binding to the H19 promoter increases H19 levels. Consequently, this expedites the G1‐S transition and facilitates cell cycle advancement [114]. The H19 RNA is involved in both MET and EMT [115]. As epithelial cells move through the extracellular matrix (ECM), blood flow eventually carries them to a susceptible site. Next, by using the opposing mechanism of metastasis, the tumor cells undergo a metastatic process that results in the formation of a secondary lesion. A linkage has been identified in regulating EMT between H19/miR‐675, Slug, and E‐cadherin (Figure 7B). According to previously, being a precursor of miR‐675 suggests that H19 may be involved in post‐transcriptional control of gene expression. The compound can inhibit c‐CbI and CbI‐b, which are two ubiquitin ligase E3s responsible for the degradation of EGFR [116, 117, 118]. Consequently, the build‐up of EGFR stimulates cell growth and movement through the Akt and Erk signaling pathways [109, 117]. It has been shown that the expression of the H19 gene is much higher in breast cancer tissues than in healthy tissues. This overexpression of the H19 gene is associated with tumor grades and the association with estrogen‐ and progesterone receptors (Figure 7B) [119].

### 5.4 GAS5

**FIGURE 8:**
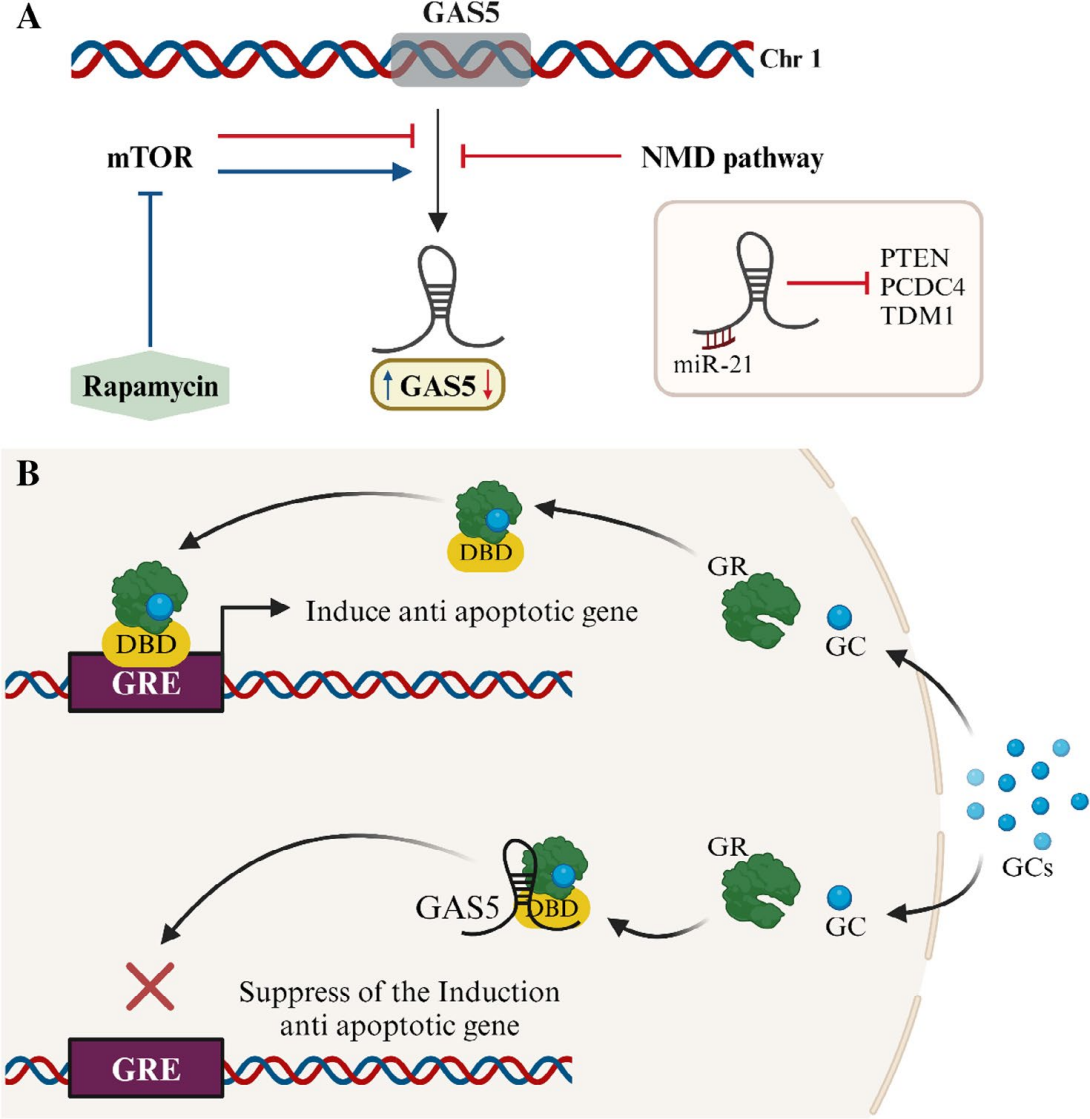
(A) When serum fasting or rapamycin induces repression of mTOR pathway activity, the translation of the GAS5 short reading frame is impeded, resulting in the cessation of nonsense‐mediated decay, which relies on active translation. The transcript level of GAS5 consequently exhibits an increase. The stimulation of mTOR signaling leads to the interaction between miR‐21 and GAS5, inhibiting GAS5 and subsequently reducing PTEN expression. The downregulation of PTEN has been seen to facilitate the migration and proliferation of cancer cells. (B) Diagram illustrating the activation of GR by glucocorticoids (GC). In GAS5 solid cancer cells undergoing downregulation, the GRs interact with the GRE via the DNA binding domain. This interaction leads to the activation of anti‐apoptotic genes, such as cIAP2, which ultimately contribute to the survival of the cells. GAS5 functions as a GRE decoy in the cells with high levels of GAS5, inhibiting the activation of anti‐apoptotic genes through GR and consequently making cells more susceptible to apoptosis.

In contrast to the previously stated long noncoding RNAs, GAS5 is considered an oncological suppressor. Its length is estimated to be between 0.6 and 1.8 kilobases, and it is located inside the Chr1q25.1 gene cluster. Its involvement affects cell proliferation, apoptosis, and the development of trastuzumab resistance [120]. GAS5 expression is significantly reduced in BC cells compared to the patients' normal breast epithelial tissue. Both stages I and II of BC exhibited decreased GAS5 expression, indicating that it should be regarded as an early occurrence in advancing the illness [121]. MTOR signaling system exhibits specific regulatory control over the translation of GAS5 [122]. Transmission occurs through mitogen‐induced translation facilitated by the p70S6K [123]. The NMD pathway represents the mechanism that decays transcripts with stop codons in the early exons; this degradation is aided by nonsense. A short‐read frame that terminates with a stop codon in exon 3 (of 12) is what causes GAS5 to be discovered. The NMD process recognizes this stop codon. GAS5 expression is decreased throughout the average period of cellular development due to the fast degradation of the GAS5 protein that occurs via the NMD pathway [124, 125]. To support this assertion, the upregulation of cellular GAS5 in different cell types, such as BC cells [122, 126], is caused by inhibiting mTOR activity by administering rapamycin. The nuclear receptor family includes the glucocorticoid receptor (GR). GR travels from the cytoplasm to the nucleus upon glucocorticoid (GC) activation. Target gene expression is regulated by GR's interactions with glucocorticoid response elements (GREs) in the nucleus [127]. The GAS5 protein suppresses the transcriptional activity that the GR produces by acting as a riborepressor of the GR. This is accomplished by connecting the GR's double‐stranded GRE mimic sequence to the bacteria's DNA binding domain [128]. The GAS5 protein competes with genuine DNA GREs to adhere to the GR (Figure 8A) [123], and it shares structural similarities with the GRE. This way, GAS5 decreases the expression of several genes, including cIAP2, making the cells prone to apoptosis [129]. Furthermore, the decrease in endogenous GAS5 levels was why BC cells resisted apoptosis [39]. Through reciprocal inhibition of miR‐21, GAS5 also functions as a suppressor [120]. MiR‐21 is overexpressed in many tumor tissues and controls malignant processes such as metastasis and cancer progression. Exon 4 of GAS5 contains a distinct binding site that allows it to act as a sponge during the interaction with miR‐21. The silencing of miR‐21 causes the transcription of other genes farther downstream to be suppressed (Figure 8B). PTEN, TDM1, and PCDC4 are some of these genes. In addition, miR‐21 can reduce GAS5 expression in BC samples by using a complex known as RISC [130, 131, 132] (Figure 8A).

### 5.5 XIST

**FIGURE 9:**
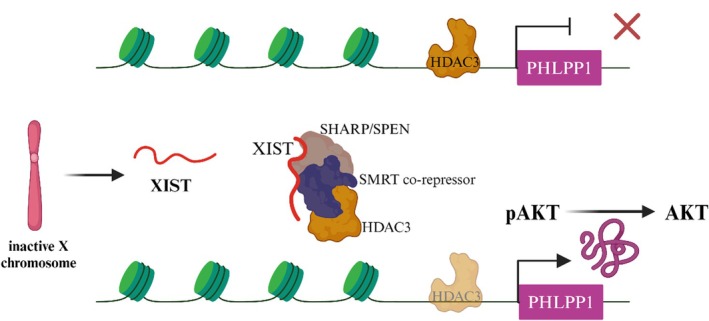
Regulatory model of XIST inhibits the activation of AKT by repressing the expression of PHLPP1 through HDAC3. XIST exerts a positive regulatory effect on PHLPP1 expression by using SPEN to sequester HDAC3 from the PHLPP1 promoter. Furthermore, PHLPP1 exerts inhibitory effects on AKT phosphorylation.

This 19‐kilobase‐long LncRNA (17‐kilobase long in mice) is called XIST, and it is essential for turning off the X chromosome during embryonic development. The function of XIST as a tumor suppressor in breast cancer has been clarified [46]. Transcribing occurs mainly on the X chromosome. Specifically, the inactive X chromosome, denoted as Xi, has a lateral distribution along this chromosome [133]. XIST is the primary factor responsible for suppressing X‐chromosome activity in female cells. X chromosome inactivation (XCI) occurs during early embryogenesis, involving the identification, selection, propagation, initiation, and maintenance of Xi chromosomes [134]. It has been discovered that females contain about a thousand suppressed genes, which results in a dosage exchange between the sexes. In female somatic cells, XIST is typically expressed. However, it has been observed that XIST expression is reduced in breast, cervical, and ovarian cancer cell lines. XIST expression is disrupted in malignancies, characterized by the absence of a conventional X chromosome, in contrast to human cells that typically have a single functional X chromosome [134, 135]. Comparing BC to normal breast tissue, the LncRNA XIST revealed a significant reduction in BC [136]. XIST functions as a ruse to indirectly control HDAC3 transcription from the promoter of the PH domain and leucine‐rich repeat phosphatase 1 (PHLPP1), with help from the co‐repressors SMRT and SHARP/SPEN. This guarantees that sufficient PHLPP1 is created. The enzyme PHLPP1 converts active pAKT into AKT, reducing cell survival. However, HDAC3, which can specifically target and block the promoter activity of PHLPP1 (Figure 9) [136], is released when XIST expression declines in BC. Studies have suggested that RNAs reduce XIST expression. Moreover, proteins like BRCA1, NANNOG, and OCT4 affect how specific processes are regulated [137]. The expression of XIST has been shown to fluctuate in cancer cells, drawing attention to the intricate and contentious function it plays in cancer biology [135].

### 5.6 bc200

bc200, also known as LncEPCAM/LncE, is elevated in nulliparous women's breast tissue and is raised in a variety of cancer types, consisting of cancer of the breast while being primarily expressed in neural cells. bc200 has promise as a molecular tool for the management of cancer of the breast, including prevention, diagnosis, screening, and prognosis. These subtypes of BC may have different phenotypes because of the downregulation of CALM2, a calcium‐binding protein involved in cell cycle development, apoptosis, and proliferation [138], which occurs as a result of bc200 overexpression. Moreover, the physiological function of this gene in nulliparous women's normal breasts may play a part in their heightened vulnerability to BC. The data highlight a number of important conclusions about bc200 in relation to BC. First off, BC tissue and cell lines both have elevated levels of bc200. It is found in the nucleus and cytoplasm of TNBC and ER+ cells. ER‐positive tumors have considerably increased expression of bc200 compared to ER‐negative tumors [139]. When bc200 is overexpressed, it has been observed to significantly boost proliferation and cell survival while also promoting invasion and cell migration. In addition, bc200 might function in cis to regulate apoptosis in TNBC and ER+ cells. Lastly, it has been shown that, in a xenograft mouse model, overexpression of bc200 promotes tumor development [47].

### 5.7 ZFAS1

ZFAS1, often referred to as HSUP1, is a 17,561 bp gene that is found on chromosome 20q13.13. It is distributed throughout the nucleus and cytoplasm fractions of cells and consists of five exons with five different transcriptional variants. ZFAS1 may interact with several transcription factors, including STAT3, KLF1, DDX4, and ZNF274. Notably, it is a very stable LncRNA that may localize in mammary tissues and control the development of epithelial cells. Its half‐life is more than 16 h. According to recent research, ZFAS1 plays a tumor‐suppressive role in BC. Using the PI3K/AKT/Phosphoinositide‐3‐kinase (PTEN)/AKT pathway, BC cells migrated, invaded, and multiplied more when ZFAS1 was downregulated [140]. It has been established that ZFAS1 interacts with STAT3, a widely recognized oncogenic transcription factor involved in the development and progression of cancer in TNBC cells. Furthermore, the function of ZFAS1 in controlling the STAT3 pathway, cellular proliferation, and spread in TNBC cells has been discovered [141]. The data show that the LncRNA ZFAS1 is highly downregulated in TNBC, a subtype of breast cancer defined by the lack of expression of HER2, the progesterone receptor, and the estrogen receptor. Its potential as a predictive biomarker is shown by the substantial correlation between its downregulation and poor survival outcomes. Functional experiments show that MDA‐MB‐231 TNBC cell clonogenicity and proliferation are enhanced when ZFAS1 is blocked. Accordingly, ZFAS1 may be involved in preventing tumor growth [142]. In addition, ZFAS1 has a significant impact on regulating the proteins associated with EMT, a process that is critical for the invasion and spread of cancer cells. The complicated regulatory mechanisms of TNBC are highlighted by the negative association observed among the STAT3 gene and ZFAS1. This suggests that the STAT3 signaling pathway may play a role in the evolution of TNBC. All of these data point to ZFAS1's importance in the pathophysiology of TNBC and suggest that it may be a useful therapeutic target [141].

### 5.8 CCAT2

In 2013, Ling and colleagues made the first discovery of CCAT2 in cancer of the colon. Subsequently, they found CCAT2 in many additional cancer types, such as cancer of the gastric, cancer of the cervical, and cancer of the breast [143, 144]. Similar to a microbiologist, the temporary increase in CCAT2 levels in T47D or MCF‐7 cells has been found to significantly inhibit cell growth and the maintenance of cell stemness. BC that tested positive for the ERα subtype had low levels of CCAT2 expression despite reports that CCAT2 promotes carcinogenesis in several cancers [145, 146]. As a regulator of the WNT signaling system, CCAT2 increases cell proliferation in culture and tumor development in living organisms [54]. It has been proven that CCAT2 levels were significantly higher in BC. Silencing CCAT2 stopped BC cells from multiplying, spreading, and going through the cell cycle in the lab and in living organisms, where it stopped tumor growth. Furthermore, via its interaction with EZH2, CCAT2 inhibits the production of p15 in cancer of the breast cells [147]. This investigation has identified a dual role of CCAT2 in controlling both tumorigenesis and the stemness of cancer cells in luminal cancer of the breast. More precisely, the cytoplasmic form of CCAT2 interacts with miRNA 221/222 to hinder cell proliferation that is reliant on p27. On the other hand, the nuclear accumulation of CCAT2 interacts with a pseudogene of OCT4 called OCT4‐PG1, which encourages the development of cancer stem cells [146].

### 5.9 SPRY4‐IT1

The SPRY4‐IT1 gene, specifically the intron region at 5q31.3, is the source of SPRY4‐IT1, a LncRNA. Firstly, it discovered the crucial involvement of SPRY4‐IT1 in melanoma apoptosis and invasion. In several cancers such as pancreatic ductal adenocarcinoma, cholangiocarcinoma, cancer of the ovary, bladder cancer, hepatocellular carcinoma, esophageal squamous cell carcinoma, and BC, SPRY4‐IT1 hinders cell death while empowering cell development and tumor proliferation [148]. However, Sun et al. discovered that overexpressing SPRY4‐IT1 stops non‐small‐cell lung cancer from growing and spreading. Research has shown that compared to MCF‐7 parental cells, CD44+/CD24‐MCF‐7 cells exhibited higher expression of SPRY4‐IT1 and that SPRY4‐IT1 promoted MCF‐7 cell self‐renewal and proliferation. The dual‐luciferase reporter research established a direct relationship between miR‐6882, SPRY4‐IT1, and TCF7L2 and demonstrated that their expression was downregulated. On the basis of these results, SPRY4‐IT1 targets miR‐6882, which increases the stemness of BC cells by regulating the activity of the Wnt/β‐catenin signaling pathway [53]. One possible contributor to the development of breast cancer is elevated SPRY4‐IT1 expression. The 8p12 amplification in luminal B cancers has been linked to zinc finger 703 (ZNF703) as the genetic driver. Recent studies in a mouse model of BC indicated that elevated ZNF703 expression was associated with an upregulation of lung metastases and a downregulation of E‐cadherin expression. The discovery that ZNF703 is a gene that SPRY4‐IT1 targets downstream and the demonstration that ZNF703 increases the growth of ER (−) breast cancer cells and inhibits apoptosis in vivo have both been made [149].

### 5.10 ANCR

ANCR is an 855‐nucleotide LncRNA that is downregulated as cells differentiate. It plays a crucial role in maintaining the epidermis's undifferentiated cell state [150]. Previous research has shown that ANCR binds to EZH2, which in turn increases its phosphorylation at Thr‐487 and Thr‐345 residues. This leads to EZH2 ubiquitination and destruction in cancer of the breast cells [15]. The process of embryogenesis and organogenesis involves the coordinated effects of tissues on TGFβ‐induced EMT, whereas ANCR suppresses cell differentiation. Given that TGF‐β and ANCR have differing impacts on differentiation, it is worth considering whether ANCR is involved in the TGF‐β signaling pathway that facilitates EMT and the spread of BC [151]. One member of the superfamily of polyomavirus enhancer‐binding proteins and core‐binding factors is runt‐related transcription factor 2, or RUNX2 [15, 152]. Cancers of the breast tissues are associated with highly elevated RUNX2 and its target genes. These play essential functions in bone metastasis [153, 154]. Because RUNX2 is increased in cells treated with TGF‐β1, it is known that RUNX2 is involved in the TGF‐β signal pathway and EMT that TGF‐β produces. Also, during osteoblast development in hFOB1.19 cells, ANCR has been shown to suppress RUNX2 expression [155]. Previous research and existing publications suggest that ANCR might be involved in the TGF‐β signal pathway and EMT produced by TGF‐β1. In addition, the study discovered that ANCR is negatively linked with RUNX2 expression in cancer of the breast tissues and many cell lines, suggesting that it may have a role in the TGF‐β signal pathway [151]. Cancer metastasis is facilitated by EZH2, an essential regulator of epigenetic modifications and an inducer of epigenetic modifications to chromatin (EMT). Several post‐translational modifications (PTMs) control EZH2 stability, and LncRNA are thought to play essential roles in various cancers. We present here the finding that ANCR regulates EZH2 stability and, by extension, cancer of the breast cell invasion and metastasis. Additionally, we find that ANCR plays a significant role in the growth and metastasis of cancer of the breast, primarily by reducing the stability of EZH2. Regarding BC in particular, we first found that ANCR levels were lower in cancerous tissues and cell lines than in cell lines and typical tissues. In light of these results, we propose a novel method for modulating EZH2 stability via ANCR‐EZH2 interaction‐promoting phosphorylation, which in turn promotes EZH2 degradation and inhibits the growth of BC [15].

### 5.11 PVT1

Murine plasmacytoma was the source of the discovery of PVT1, an oncogenic LncRNA measuring 300 kilobases and situated on human chromosome 8q24, 57 kilobases downstream of the c‐MYC locus. Many malignancies have an overexpression of PVT1, which indicates its function as an oncogene in carcinogenesis [156]. There is strong evidence that PVT1 promotes the growth and spread of cancer of the breast in both laboratory and animal studies. PVT1 is also substantially expressed in plasma from BC patients and cells. PVT1 expression significantly increases in cancer of the breast patients' cell lines and plasma. The process by which PVT1 promotes epithelial‐mesenchymal transition involves upregulating FOXQ1 via miR‐128‐3p. Furthermore, PVT1 promotes BC cell proliferation, metastasis, and epithelial‐mesenchymal transition via its binding to the UPF1 protein [157]. Metastasis, EMT, and modulation of apoptosis are only a few of the clinicopathological features of BC linked to PVT1 amplification. Furthermore, transcripts produced from PVT1 may potentially contribute to the development of breast tumors [158, 159, 160]. Also, PVT1 modulates transcription factors known to have oncogenic roles in cancer, which increases breast tumorigenicity [161]. Studies have shown that PVT1 can promote cell growth and prevent cell death in ovarian and cancer of the breast cells. Additionally, it has been found to play a role in regulating the response to chemotherapy in pancreatic cancer. Moreover, it has been shown that PVT1 increases the expression of PAI‐1, plasminogen activator inhibitor‐1, which promotes angiogenesis, invasion, and cell migration [162]. According to research, the LncRNA PVT1 is essential for the growth of tumors in TNBC by enhancing the KLF5/beta‐catenin signaling pathway. An increase in PVT1 expression in TNBC tumors is seen in a clinical setting. Genetic investigations targeting PVT1 in TNBC cells revealed that PVT1 depletion resulted in a noteworthy decrease in colony formation, cell proliferation, and the development of orthotopic xenograft tumors. From a biological perspective, PVT1 interacts with KLF5 and enhances its stability through BAP1, leading to the upregulation of beta‐catenin signaling. This ultimately contributes to the promotion of TNBC tumorigenesis. Clinical TNBC samples co‐expressed KLF5, beta‐catenin, and PVT1 [76]. In BC, it has been discovered that PVT1 promotes cell proliferation and inhibits apoptosis. Epigenetic mechanisms involving EZH2 accomplish the suppression of FOXF1 expression [163].

### 5.12 UCA1

UCA1 is a LncRNA that consists of three exons, giving rise to two isoforms—one measuring 1.4 kb and the other measuring 2.2 kb. According to some recent research, the 1.4 kb isoform of UCA1 may function as an oncogene in BC. In short, UCA1 may lower the amount of p27 protein via competitive interaction with hnRNP I, which promotes the development of BC. Furthermore, it can enhance the growth of BC cells and inhibit cell death by reducing the levels of tumor‐suppressive miR‐143. Recent research has shown that the activation of UCA1 can enhance the invasion of BC cells. Nevertheless, the underlying mechanism remains somewhat elusive [58]. In ER‐positive cancer of the breast cells, the researchers discovered that tamoxifen causes an increase in UCA1 expression, and this effect is dependent on HIF1α. Increased expression of UCA1 leads to a notable increase in resistance to tamoxifen. UCA1 sponges miR‐18a, which negatively regulates HIF1α. Thus, the upregulation of UCA1 is further strengthened by a feedback loop involving miR‐18a and HIF1α [164]. An actual tumor suppressor, ARID1A, works with p53 to control the transcription of SMAD3 and CDKN1A, as well as the formation of tumors in gynecologic malignancies. Notably, ARID1A controlled the transcription factor CEBPα's chromatin access, which in turn suppressed the production of a LncRNA called UCA1. Restoration tests demonstrated that UCA1 mediates ARID1A's ability to cause a loss of cellular migration and proliferation. Collectively, our findings define ARID1A as a crucial tumor‐suppressor gene in BC via its collaboration with CEBPα. Mutations resulting in loss of function of ARID1A trigger the activation of UCA1 [165]. TGF‐β caused alterations in the amounts of LncRNAs across the genome in cancer of the breast cells. Among them, AC026904.1 and UCA1 exhibited elevated expression in metastatic cancer of the breast and were strongly linked to a worse prognosis. According to a mechanistic analysis, the conventional and noncanonical TGF‐β pathways, respectively, were responsible for the upregulation of UCA1 and AC026904.1. Subsequent investigation revealed that whereas UCA1 operates as ceRNA in the cytoplasm, AC026904.1 serves as an enhancer RNA in the nucleus. Furthermore, at both the transcriptional and post‐transcriptional stages, AC026904.1 and UCA1 might work together to enhance Slug expression, playing crucial roles in the EMT produced by TGF‐β [166].

### 5.13 LINP1

It has been revealed that chromosome 10's LncRNA LINP1 (LINP1) is up‐regulated in cancer of the breast tissues and contributes to the disease's development. Previous research has shown LINP1's carcinogenic impact [167]. It is now well established that LINP1 suppresses cell proliferation and metastasis in order to mediate its oncogenic involvement in BC. Furthermore, LINP1 overexpression was linked to chemoresistance and was positively correlated with 5FU‐ and doxorubicin (DOX)‐resistant breast tumor cell lines. Upregulated LINP1 expression in cancer of the breast was also linked to a poor prognosis [168]. Furthermore, by acting as a scaffold between DNA‐PKcs and Ku80 and directing the NHEJ process, LINP1 improves double‐strand DNA break repair. Significantly, in BC, inhibiting LINP1, which is controlled by p53 and EGFR signaling, enhances the tumor cell's susceptibility to radiation treatment [169].

### 5.14 SRA

In a groundbreaking discovery in 1999, the presence of SRA, a LncRNA with the unique ability to co‐activate steroid nuclear receptors, was first reported. This finding not only expanded our knowledge of LncRNAs but also shed light on their role in enhancing steroid receptor transcriptional transactivation when SRA RNAs are overexpressed. SRA, a LncRNA with multiple isoforms, was initially identified as a functional LncRNA that primarily stimulates the transcriptional activity of steroid receptors, a family of RNAs that continues to grow. Located on locus 5q31.3, it is composed of four introns and five exons [61]. SRA transcripts of approximately 0.7–0.9 kb in length are the most common in normal tissue; longer, less common transcripts (1.3–1.5 kb) have also been found [170]. The gene SRA1 has two roles: first, it is an RNA activator; second, it produces a conserved protein called SRAP, which may influence gene transcription in ways that are particular to cells and systems. Studies using gain–loss‐of‐function methods have shown that SRA1 products are essential for normal zebrafish heart development and have a significant impact on BC invasion. In contrast, SRAP inhibits the transcriptional regulatory activity of the SRA1 gene by binding a particular SRA stem loop [171]. SRA may boost steroid receptor transcriptional activity on target genes while also acting as a unique scaffold. It was previously discovered that the disruption of SRA1 has a significant impact on a repressive complex, causing various proteins necessary for its repressive function to come together. This finding further supports the idea that SRA1 acts as a scaffold in cancer of the breast cells [172]. There is an elevated level of SRA in tumors of the breast, and its expression is linked to receptors of estrogen and receptor of progesterone levels. This correlation could impact ER/PR action and contribute to the development of tumors [61]. In addition, specific signaling pathways, including Notch, p38 MAPK, and TNFα pathways, have been found to be crucial in the development of estrogen‐dependent cancer of the breast. Furthermore, the latest data reveals that the expression of SRA could potentially function as a novel prognostic indicator in individuals diagnosed with ER‐positive cancer of the breast [173].

### 5.15 LINK‐A

Research on cancer has made LINK‐A, which is also referred to as LINC01139, a major LncRNA. It is situated on chromosome 1q43. It consists of two exons and covers a length of 5634 nucleotides. Discovered in 2006, this LncRNA, known as LINK‐A, measures approximately 1.5 kb. LINK‐A shows increased expression in various tumor samples, such as cancer of the breast, cancer of the ovary, glioma, mantle cell lymphoma, cancer of the non‐small‐cell lung. It is worth mentioning that LINK‐A plays a role in controlling important pathways related to cancer, such as HIF1α and AKT signaling. It is also associated with various cancer‐promoting activities, including apoptosis, cell proliferation, EMT, migration, cell invasion, and glycolysis reprogramming [174]. According to research by Lin et al., normoxic HIF1α signaling is activated by LINK‐A and is important in tumor formation, especially in TNBC. LINK‐A takes part in the process of signal transduction directly. In order to phosphorylate and stabilize HIF1α, LINK‐A recruits and activates BRK kinase, which in turn causes HIF1α to be phosphorylated. HIF1α signaling is essential for cancer cells to adapt to hypoxia (low oxygen levels) and for tumor growth, metastasis, and glycolysis reprogramming [62]. Additionally, the AKT pathway is hyperactivated as a result of LINK‐A's interaction with PtdIns P3, a lipid molecule that is implicated in the pathways of cell signaling. This relationship makes cancer of the breast cells more resistant to AKT inhibitors such as MK2206 and Perifosine, while also increasing their survival and proliferation [175]. In different studies, LINK‐A was shown to be an oncogenic LncRNA. It was found to downregulate intrinsic tumor suppression pathways and cancer cell antigen presentation. This immune evasion pathway was explored by utilizing many cancers of the breast cell lines and a transgenic animal model. The research revealed LINK‐A's involvement in the cAMP/PKA pathway and GPCR‐PKATRIM71 signaling, which support tumor development, metastasis, immunosuppression, and resistance to immunotherapy. These data, taken together, underscore LINK‐A's critical involvement in driving BC growth and development [62, 176].

## 6 LncRNA: Properties, Distribution, and Research Tools

At least 98% of human transcripts are translated into noncoding RNA [177], divided into two main categories. Only around 2% of transcripts are translated into proteins. Short noncoding RNAs comprise the first group, including siRNAs, piRNAs, miRNAs, and snoRNAs. LncRNAs make up the second category [5]. LncRNAs are particular RNA molecules that are longer than 200 nt and do not code for proteins produced by the RNApolII enzyme. A polyA tail is present at the 3′ terminus of approximately half of the spliced RNA molecules with a 5′ cap structure. Protein encoding is one of the characteristics that sets LncRNAs apart from mRNA [178]. Messenger, transfer, ribosomal, and circular RNAs are transferred out of the cytoplasm. However, some LncRNAs and circular RNAs stay in the nucleus, while snRNAs are processed in the cytoplasm before being imported back into the nucleus [179]. LncRNAs can act in the cytoplasm or the nucleus, occasionally in both. While LncRNAs in the cytoplasm interact with mature messenger RNA and proteins, their counterparts in the nucleus mainly function to control transcription, remodel and change chromatin, or process RNA [180].

Because LncRNAs typically lack polyA tails and have low expression levels, detecting and examining them using conventional techniques like mRNA‐seq and microarray can be challenging. The foundation of these techniques is isolating polyA+ transcripts, which are more likely to be highly expressed. Notwithstanding these drawbacks, several effective methods for identifying and annotating LncRNAs have been developed [181]. The study of LncRNAs has benefited tremendously from the use of conventional methods. Current research is developing many innovative RNA‐focused approaches. The primary topics are the structure, interaction partners, and placement of LncRNAs from these methods [182]. In order to discover novel LncRNAs, RNA‐seq is now routinely utilized. Sequencing is becoming less expensive, and RNA‐seq offers accurate nucleotide identification [181]. Due to the low expression levels of LncRNAs, finding these rare LncRNAs requires large sequencing depths (at least 100–150 million reads). LncRNAs are found by RNA‐seq investigations, which require either decreasing rRNA to enrich for mRNAs and LncRNAs or sequencing both polyA+ and polyA‐fractions [183]. An extensive range of experimental techniques is accessible for LncRNA annotation. The approach known as RNA immunoprecipitation‐sequencing, or RIP‐Seq, allows the sequencing of RNAs associated with a specific RNA‐binding protein by immunoprecipitation [184]. An approach similar to this is known as chromatin isolation by RNA purification (ChIRP)‐Seq. It involves making a large number of oligonucleotide probes tagged with biotin that are capable of identifying a particular transcript. After this, cross‐linked chromatin complexes containing the target RNA are separated using streptavidin magnetic beads sensitive to the biotin tag [185, 186]. RNA‐FISH is an additional approach in which fluorescent probes for RNA are created; the probes are then hybridized in cells and imaged under a microscope. When studying how LncRNA localization varies in reaction to stimuli or when examining LncRNA placements in various cellular compartments, this method works well [185]. RNA‐3C makes use of biotinylated oligonucleotides to aid in the synthesis of double‐stranded cDNA. This process aims to obtain chromatin complexes containing biotinylated cDNA, which is then digested and proximity ligated to produce DNA‐cDNA molecules. The biotin group can then extract these structures, and PCR can be used to analyze them. This process aims to obtain chromatin complexes containing biotinylated cDNA, which is then digested and proximity ligated to produce DNA‐cDNA molecules. The biotin group can then extract these structures, which PCR can analyze. RNA‐3C has been utilized to explore the relationships between LncRNAs and the three‐dimensional organization of the genome [187]. Several ways can be used to modify the expression levels of LncRNA, one of which is to use CRISPR to change the genomes of LncRNA genes [188]. Furthermore, CRISPR can interfere with nuclear and cytoplasmic LncRNAs using innovative antisense oligonucleotide (ASO) techniques [181].

## 7 Conclusions and Future Perspectives

The complex process of metastasis involves the participation of a significant number of different molecular pathways. With the knowledge we now possess about the signaling pathways and the molecular and cellular processes responsible for the spread of cancer, we are able to identify potential targets for intervention. Following the occurrence of these improvements, the process of determining intervention targets has become less complicated. Because they regulate a large number of cellular signaling pathways, LncRNA allows us to influence a wide range of biological mechanisms. The malfunctioning of LncRNA has been connected to a number of human diseases, including cancer. Several cellular signaling pathways might be affected by inadequate control of LncRNA. These pathways affect various processes, including cellular growth, death, and inflammation. Both the beginning and the progression of cancer are closely linked to a number of different pathways, each of which makes a direct contribution to the two processes when they are considered separately. The specific function of LncRNA in the development of cancer is yet unclear, despite the fact that an increasing quantity of research is being conducted to investigate this topic. On the other hand, LncRNA provides a considerable contribution to the process of cancer metastasis, which is a significant contributor to the mortality rate of cancer patients. Furthermore, in addition to interacting with nucleotide sequences, LncRNA is also capable of interacting with proteins. As a result, they are now able to embrace more complicated frameworks. Compared to microRNAs, they are capable of carrying out movements that exhibit a higher level of complexity. LncRNA has been shown to affect a wide variety of biological processes that are connected with BC, including angiogenesis, metastasis, proliferation, and treatment resistance. The advancement of RNA‐centric techniques will lead to an improvement in our understanding of LncRNA, which will be of assistance in the prevention, detection, and treatment of borderline personality BC. The material that has been provided is a big step forward in the fight against BC. More studies are required to acquire a comprehensive understanding of the function that cancer‐specific LncRNA plays in the development of cancer metastasis and the underlying mechanisms that they use. The potential incorporation of LncRNA biology into the study of cancer biology is a possible approach that might provide us with a more profound knowledge of the processes that are responsible for the spread of cancer. This study explores the biological characteristics of LncRNAs to understand their role in regulating cell signaling pathways and alterations in cell survival, proliferation, and invasion elements that distinguish BC. It is a prevalent disease affecting women worldwide, and the development of new biomarkers can help diagnose and predict its course. LncRNAs play a crucial role in BC development through various biochemical mechanisms. Aberrant LncRNA expression significantly impacts BC progression by interacting with NF‐κB, TGF‐β, p53, EGF, and PI3K/AKT pathways. In addition to this, it has the potential to offer new avenues for targeted, effective, and practical therapeutic and diagnostic procedures. In addition, a proposition has been put out that suggests that the intentional targeting of LncRNA could impede the advancement of carcinogenesis to a greater degree. More excellent studies are advised to explain the functions that LncRNAs play in BC; nonetheless, new clinical trials may bring forth unique treatment possibilities.

## Author Contributions

Study concept and design: S.G., R.M., and A.A; analysis and interpretation of data: S.G. and R.M.; drafting of the manuscript: A.A. and R.M.; critical revision of the manuscript for important intellectual content: S.G.

## Funding

The authors have nothing to report.

## Conflicts of Interest

The authors declare no conflicts of interest.

## Data Availability

Data sharing not applicable to this article as no datasets were generated or analyzed during the current study.
